# Calcitonin gene-related peptide: a potential protective agent in cerebral ischemia–reperfusion injury

**DOI:** 10.3389/fnins.2023.1184766

**Published:** 2023-07-17

**Authors:** Jie Xiong, Zhiyong Wang, Junhui Bai, Keling Cheng, Qicai Liu, Jun Ni

**Affiliations:** ^1^Department of Rehabilitation, The First Affiliated Hospital of Fujian Medical University, Fuzhou, Fujian, China; ^2^Department of Reproductive Medicine Centre, The First Affiliated Hospital of Fujian Medical University, Fuzhou, China

**Keywords:** ischemia–reperfusion injury, brain, apoptosis, calcitonin gene-related peptide, nerve damage repair

## Abstract

Ischemic stroke is the most common type of cerebrovascular disease with high disability and mortality rates, which severely burdens patients, their families, and society. At present, thrombolytic therapy is mainly used for the treatment of ischemic strokes. Even though it can achieve a good effect, thrombolytic recanalization can cause reperfusion injury. Calcitonin gene-related peptide (CGRP) is a neuropeptide that plays a neuroprotective role in the process of ischemia–reperfusion injury. By combining with its specific receptors, CGRP can induce vasodilation of local cerebral ischemia by directly activating the cAMP–PKA pathway in vascular smooth muscle cells and by indirectly activating the NO–cGMP pathway in an endothelial cell-dependent manner,thus rapidly increasing ischemic local blood flow together with reperfusion. CGRP, as a key effector molecule of neurogenic inflammation, can reduce the activation of microglia, downregulates Th1 classical inflammation, and reduce the production of TNF-α, IL-2, and IFN-γ and the innate immune response of macrophages, leading to the reduction of inflammatory factors. CGRP can reduce the overexpression of the aquaporin-4 (AQP-4) protein and its mRNA in the cerebral ischemic junction, and play a role in reducing cerebral edema. CGRP can protect endothelial cells from angiotensin II by reducing the production of oxidants and protecting antioxidant defense. Furthermore, CGRP-upregulated eNOS can further induce VEGF expression, which then promotes the survival and angiogenesis of vascular endothelial cells. CGRP can also reduce apoptosis by promoting the expression of Bcl-2 and inhibiting the expression of caspase-3. These effects suggest that CGRP can reduce brain injury and repair damaged nerve function. In this review, we focused on the role of CGRP in cerebral ischemia–reperfusion injury.

## 1. Introduction

Ischemic stroke is a type of stroke that accounts for 71% of all stroke subtypes and is the most common type of cerebrovascular disease (Tuo et al., 2022). Ischemic stroke is mainly caused by a thrombus or an embolus blocking the main cerebral artery or by a spasm of the main cerebral artery. It leads to the severe interruption of blood flow in the ischemic area of the brain, which then causes brain tissue death with high disability and mortality rates (Wang W. et al., 2017; Fan et al., 2020), creating severe economic and social burden to patients, their families, and society. Ischemic stroke includes a severely ischemic central infarction and the penumbra around the infarction. The nerve cells in the infarction area usually die, whereas brain tissues in the penumbra are impaired but remain alive (Liu et al., 2011). Precisely due to the existence of the penumbra after the occurrence of cerebral ischemia, treatment should be provided immediately to restore the local cerebral blood flow and prevent the deterioration and necrosis of the penumbra. At present, thrombolytic therapy is mainly used in the treatment of ischemic stroke; although it can achieve a good effect, its therapeutic time window is considerably narrow, and reperfusion injury may occur after thrombolysis and recanalization, resulting in secondary brain injury, which causes some limitations (Raedschelders et al., 2012). Early intervention of ischemia–reperfusion injury and the development of drugs that promote the brain tissue repair of cerebral ischemia–reperfusion injury are of great significance for the treatment of ischemic stroke. Therefore, new treatment models or intervention strategies for the effective treatment of ischemic strokes should be developed.

Calcitonin gene-related peptide (CGRP) is a neuropeptide composed of 37 amino acids. There is a deal of evidence that it is important for CGRP to maintain cardio-cerebrovascular homeostasis under physiological conditions, and CGRP may play a role in vasodilation during cerebral and cardiac ischemia (de Boer et al., 2020). Some studies have shown that in the acute phase of subarachnoid hemorrhage (SAH), the contraction of cerebral artery to endothelin-1 and 5-hydroxytryptamine increases, and the vasoconstriction induced by depolarization is significantly increased, while the secretion of endogenous CGRP into cerebrospinal fluid (CSF) is considered to have a protective effect on cerebral ischemia associated with vasospasm (Bründl et al., 2021; Li et al., 2021). Since CGRP-mediated relaxation is an important balance to enhance arterial contractility, the decrease of CGRP release after SAH will aggravate vasospasm after SAH (Johansson et al., 2019). CGRP can also reduce angiotensin II (AngII)-induced hypertension, thus preventing hypertension (Sabharwal et al., 2019). In the brains of people with diabetes, high blood sugar can damage the cerebrovascular system as well as neurovessels. Some studies have shown that, in contrary to promoting angiogenesis under normal glucose, CGRP inhibits hyperglycemia-induced tubule formation; CGRP also inhibits apoptosis and partially reduces the increase of intracellular reactive oxygen species (ROS) (Guo et al., 2019). These findings prove the protective effect of CGRP overexpression on high glucose-induced cerebrovascular changes. At present, monoclonal antibodies (McAb) against CGRP system have been proved to be effective, safe and well tolerated in reducing migraine attacks (Ogunlaja and Goadsby, 2022). Although CGRP McAb seems to be safe, in routine clinical practice, a 41-year-old woman with non-aura migraine developed right thalamic infarction after taking the first dose of CGRP McAb (Aradi et al., 2019); and clinically, a migraine patient treated with CGRP McAb developed reversible cerebral vasoconstriction syndrome (Rozen and Bhatt, 2022). Therefore, before using CGRP monoclonal antibody in migraine patients, it is necessary to evaluate whether there is a potential factor for cerebral ischemia; for patients with increased risk of stroke, CGRP McAb should be used cautiously. At the same time, CGRP also can prevent ischemia–reperfusion injury and improve the outcome after an ischemic stroke (Liu et al., 2011). After cerebral ischemia–reperfusion, the expression of CGRP changes in the injured site, and the degree of CGRP expression is related to the repair of nerve injury (Bucinskaite et al., 1998). This article reviews the mechanism of nerve repair of CGRP in cerebral ischemia–reperfusion injury, aiming to reveal new neuroprotective intervention measures.

## 2. Distribution and structure of CGRP

CGRP is a 37-amino acid (5 KDa) neuropeptide, with two α and β subtypes; α-CGRP is produced by the selective splicing of the calcitonin gene, and β-CGRP is encoded by isolated genes (Amara et al., 1985; Russell et al., 2014). The difference between α-CGRP and β-CGRP in humans is the presence of three amino acid residues, whereas only a one-amino acid residue difference is found between them in rats. Even though these two forms of CGRP are different, they have similar biological effects (Sexton, 1991). CGRP—as a neuropeptide—is widely expressed in the central and peripheral nervous systems and is distributed in the gastrointestinal tract, muscles, cardiovascular tissues, and other tissues and organs along with the peripheral nerve (Brain and Grant, 2004; De Col et al., 2018; Hendrikse et al., 2019). The synthesis and release of CGRP are regulated by the activity of the transient potential receptor (TRP), which has many subtypes, such as TRPV, TRPA, TRPC, and so on. Among them, TRPV1, which is also known as the capsaicin receptor or vanillin receptor 1, is the most widely studied receptor that promotes the cell secretion of CGRP; it can be activated by capsaicin, high temperature (> 43°C), low PH, and other endogenous substances (Edvinsson et al., 1990; Geppetti et al., 1991; Kessler et al., 1999). The biological function of CGRP is achieved by binding to its receptors. The CGRP receptor is a type of heterodimer that belongs to the G protein-coupled receptor family. It is composed of a calcitonin receptor-like receptor (CRLR), receptor activity modification protein 1 (RAMP1), and receptor component protein (RCP). Individually, CRLR has no biological role in regulating CGRP-induced cellular function, and its receptor activity depends on binding to RAMP1. As a key receptor subunit of CGRP, RAMP1 is similar to a molecular chaperone that transports CRLR to the cell surface; at the same time, RCP, as a receptor subunit coupling the CGRP receptor and its downstream signal pathway, is also essential for the binding of CGRP to specific receptors (Lennerz et al., 2008). In the periphery, CGRP plays a series of different roles, including dilating blood vessels, relaxing smooth muscles, reducing gastric acid secretion, protecting gastric mucosa, and directly stimulating cardiac contractility and contractile rate (Sexton, 1991). In the central nervous system, CGRP exhibits anti-inflammatory and anti-apoptotic effects, promotes nerve repair and angiogenesis, and protects neurological function (Borkum, 2019).

## 3. Changes in CGRP expression in the brain after cerebral ischemia–reperfusion

CGRP is widely expressed in the central and peripheral nervous systems and is distributed in the gastrointestinal tract, muscles, cardiovascular tissues, and other tissues and organs along with the peripheral nerve. As the strongest vasodilator, CGRP can dilate the blood vessels of ischemic and hypoxic tissues, restore the blood supply of ischemic tissues, and reduce tissue injury. Furthermore, CGRP plays a neuroprotective role during cerebral ischemia–reperfusion injury. After cerebral ischemia, the concentration of CGRP in tissues around the ischemia increases, and the survival of ischemic tissue is positively correlated with CGRP concentration (Bucinskaite et al., 1998; Bulloch et al., 1998). This finding suggests that CGRP has pre-adaptation and anti-reperfusion injury effects and that CGRP expression may be an important protective component of the nervous system response to injury. After cerebral ischemia–reperfusion, the expression of CGRP in peri-ischemic tissue further increases (Lei et al., 2000), and ischemia–reperfusion can upregulate the expression of CGRP receptor CRLR (Zhao et al., 2016). Zhang et al. (2011) found that after cerebral ischemia–reperfusion, the expression of CGRP in neurons around the ischemia increases; however, the overall level in the brain is significantly lower than that in the sham-operated group, which is not conducive in repairing the injured tissue. Studies have shown that exogenous CGRP can significantly increase the concentration of CGRP in the brain, reduce neuronal apoptosis and nervous system injury, and maintain the survival of neurons. Consistent with the results of Du et al. (2018) also found that the overall level of CGRP in the brain after cerebral ischemia–reperfusion is significantly lower than that in the sham operation group, and exogenous CGRP treatment can significantly upregulate the expression of vascular endothelial growth factor (VEGF) and basic fibroblast growth factor (bFGF), reduce infarct size, inhibit apoptosis, and increase the number of surviving neurons. Furthermore, promoting the release of CGRP or increasing the concentration of CGRP in brain tissues plays a positive role in alleviating brain injury and promoting neuronal survival.

## 4. Protective factors of CGRP in ischemia–reperfusion injury

### 4.1. Ischemia–reperfusion injury-induced apoptosis

Apoptosis is the main cause of brain death after cerebral ischemia–reperfusion. First, ischemia leads to the destruction of the oxidative phosphorylation of the mitochondrial inner membrane, resulting in ion imbalance, energy depletion, cell membrane depolarization, excessive calcium ion (Ca2+), and the accumulation of extracellular glutamate (excitatory amino acids; Sims and Muyderman, 2010). A large amount of extracellular glutamate is toxic and may cause neuronal death. In high extracellular glutamate levels, N-methyl-D-aspartic acid (NMDA) and metabotropic glutamate receptors and α-amino-3-hydroxy-5-methyl-4-isoxazole-propionic acid (AMPA) and variable glutamate receptors are activated; the activation of glutamate receptors leads to calcium overload, which then activates calcium-dependent enzymes, reactive oxygen species (ROS), and cell death pathways. Excessive release of calcium and mitochondrial potential leads to the accumulation of metabolic wastes and tissue damage (Wang F. et al., 2017). Mitochondria play an important role in the production of ROS in cells (Niizuma et al., 2010). After cerebral ischemia, the balance between ROS clearance and production is disrupted, resulting in signal transduction and cell injury induced by oxidative stress, and severe oxidative stress can lead to cell death through necrotic or apoptotic pathways (Karamyan, 2021). When the blood supply to the ischemic tissue is restored, the reperfusion injury may be more destructive than the initial ischemia because restoring blood flow replenishes oxygen to the tissue and increases the production of oxygen free radicals that can damage cells; in addition, the reinfusion of blood flow aggravates the inflammatory response of the damaged tissue, prompting leukocytes to kill damaged cells that can otherwise survive (Raedschelders et al., 2012).

### 4.2. Upregulation of B-cell lymphoma 2 (bcl-2) and inhibition of caspase-3 expression by CGRP

After cerebral ischemia–reperfusion, the expression of activated caspase-3 in the brain increases and promotes apoptosis. Caspase-3 protease is a key mediator of apoptosis during cerebral ischemia–reperfusion, and it can be activated by various factors to promote apoptosis (Yu et al., 2016). In the central nervous system, the expression of caspase-3 is regulated by inducible nitric oxide (NO) synthase (iNOS), which induces caspase-3-mediated apoptosis in neural cells (Luo et al., 2016). Studies have shown that CGRP treatment can effectively inhibit caspase-3 activity and apoptosis and downregulate the expression of apoptosis-related genes, including caspase-3, caspase-8, caspase-9, and Bax (Song et al., 2009; Wu et al., 2018). As another important regulator of apoptosis, bcl-2 plays an important role in regulating apoptosis. It can prevent the release of cytochrome C from the mitochondria to the cytoplasm, thus inhibiting apoptosis (Wang et al., 2016). The expression of bcl-2 is regulated by cyclic adenosine monophosphate (cAMP) response element binding protein (CREB). As a transcriptional factor or co-transcriptional factor expressed constitutively in neurons, CREB regulates the expression of genes containing the cAMP-response element in the promoter region, such as the bcl-2 gene. CREB is the junction of multiple signaling pathways in cells and plays an important role in mediating neuronal growth or survival, synaptic plasticity, and neuroprotection (Sugiura et al., 2004). After phosphorylation, p-CREB binds to the cAMP response elements on specific DNA and binds to the CREB-binding protein (CBP). The CBP bound to CREB acts on general transcription factors, such as TFII D, promotes the binding of general transcription factors to gene promoters, and activates gene transcription, thus expressing the bcl-2 protein (Zheng et al., 2019). After cerebral ischemia–reperfusion, the expression of activated caspase-3 in the brain increases, and at the same time, the level of phosphorylated CREB increases correspondingly to resist apoptosis; however, the level of CREB in the brain decreases significantly, which is not conducive in inhibiting apoptosis (Zhang et al., 2010; Yu et al., 2016). CGRP can up-regulate the expression of CREB and activate CREB through a series of signaling pathways, up-regulate the level of phosphorylated CREB, and then increase the expression level of bcl-2 (Zhang et al., 2010). Furthermore, CGRP can also reduce the expression of caspase-3 by inhibiting the expression of Inos (Luo et al., 2016), thus activating the anti-apoptosis pathway. However, treatment with CGRP8-37, which is an RAMP1 and 2 inhibitor, eliminates the protective effects of CGRP (Sueur et al., 2005; Lei et al., 2016); these data further suggest that CGRP exhibits anti-apoptotic properties. In addition, CGRP can prevent excitotoxicity, apoptosis, and cytolysis by buffering intracellular Ca2+, reducing the activity of NMDA receptors, and releasing glutamate, thus reducing brain death and achieving neuroprotection (Sakamoto et al., 2014; Zhou et al., 2015).

## 5. Protective mechanism of CGRP against cerebral ischemia–reperfusion injury

### 5.1. Vasodilation properties of CGRP in ischemia–reperfusion injury

CGRP is one of the strongest vasodilators currently found. Its dilation intensity is 10 times stronger than that of prostaglandins and 100–1,000 times more than that of other common vasodilators, such as acetylcholine, 5-HT, substance P, and so on (Brain and Grant, 2004). After cerebral ischemia–reperfusion, the level of CGRP in neurons in brain tissue increases accordingly (Bucinskaite et al., 1998). CGRP binds to and activates the receptors on vascular smooth muscle cells, and the activated receptor complex further activates adenylate cyclase, resulting in the increase and accumulation of cAMP in smooth muscle cells (Brain and Grant, 2004; Russell et al., 2014). The accumulation of cAMP further activates protein kinase A (PKA), which then phosphorylates various downstream factors, including the ATP-sensitive potassium channel (KATP) and large conductance Ca2 + −activated potassium channel (KCa), which hyperpolarizes the vascular smooth muscle cell membrane to inhibit Ca2+ influx (Quayle et al., 1994; Miyoshi and Nakaya, 1995; Wellman et al., 1998). Studies have shown that CGRP can also activate the sarcoplasmic reticulum to uptake intracellular Ca2+ by activating the calcium pump ATP enzyme, thus transferring intracellular Ca2+ to the storage site, which further reduces the concentration of Ca2+ in smooth muscle cells (Kimura et al., 1982; Raeymaekers et al., 1990). The hyperpolarization of smooth muscle cells and the decrease of intracellular Ca2+ concentration lead to the relaxation of vascular smooth muscle.

At the same time, CGRP can also bind to receptors on endothelial cells and stimulate the formation of cAMP in endothelial cells, and the catalytic subunit of cAMP further phosphorylates and activates endothelial NO synthase (eNOS), resulting in increased synthesis and release of NO (Crossman et al., 1990; Butt et al., 2000). NO diffuses to vascular smooth muscle cells, which then activates guanylate cyclase in smooth muscle cells, resulting in the production and accumulation of cyclic guanosine monophosphate (cGMP; Akerman et al., 2002). The activation of the NO-cGMP pathway leads to the activation of KATP and KCa channels, the blockade of calcium channels, and a decrease in Ca2+ concentration, ultimately leading to vasodilation (Rosenfeld et al., 2000; Pimentel et al., 2007; Qu et al., 2014).

**Figure 1:**
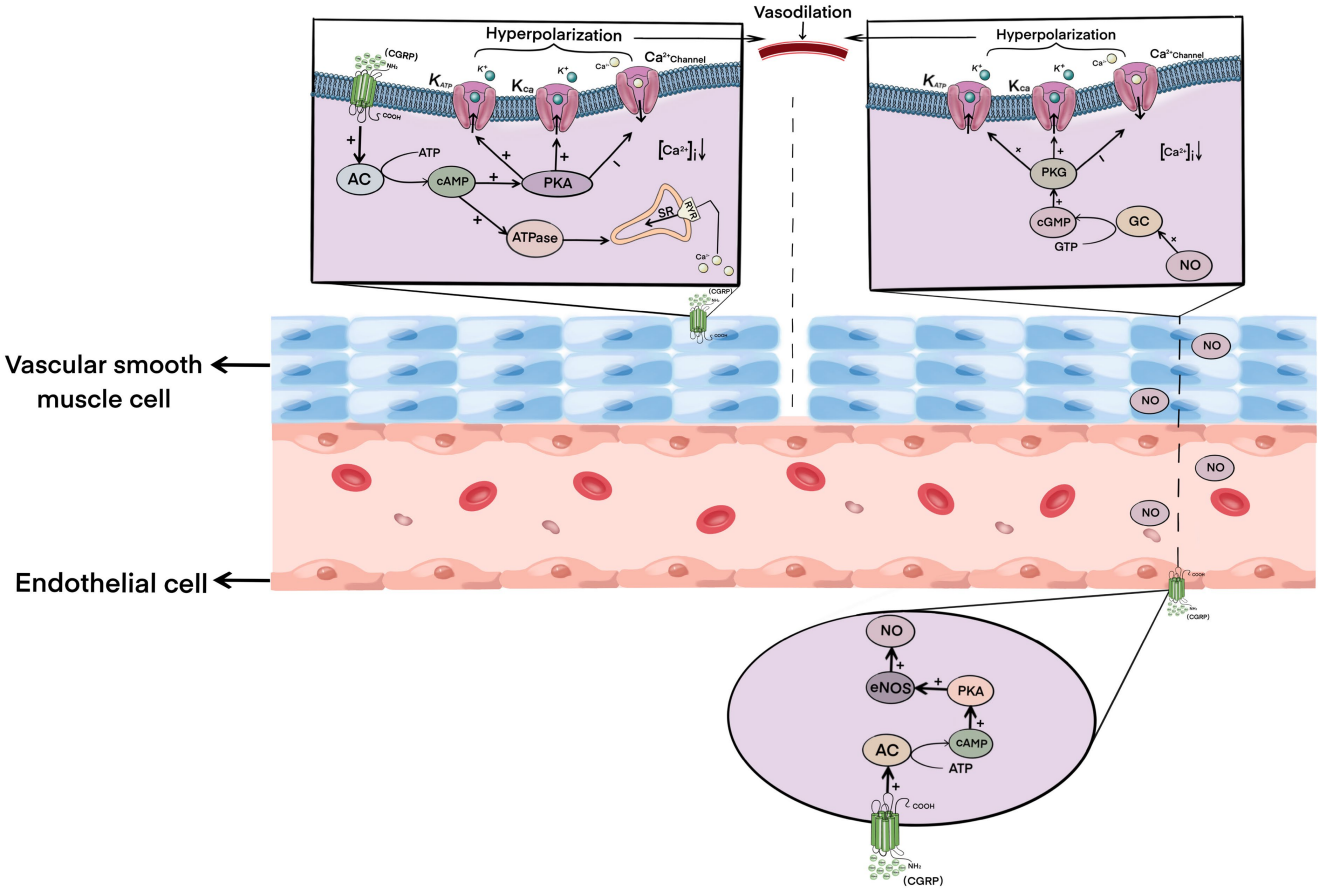
Mechanism of calcitonin gene-related peptide-induced vasodilation.

In conclusion, CGRP can induce vasodilation of local cerebral ischemia by directly activating the cAMP–PKA pathway in vascular smooth muscle cells and by indirectly activating the NO–cGMP pathway in an endothelial cell-dependent manner (Figure 1), thus rapidly increasing ischemic local blood flow together with reperfusion, cooperatively protecting the brain tissue of ischemic penumbra by ischemia and hypoxia-induced damage, and reducing infarct size.

### 5.2. CGRP and cerebral edema

Brain edema is the main cause of stroke morbidity and mortality (Frydenlund et al., 2006). Studies have shown that ROS are excessively produced during cerebral ischemia–reperfusion, which causes oxidative damage to vascular endothelial cells, thus destroying the blood–brain barrier and leading to brain edema (Nagel et al., 2008). The blood–brain barrier is composed of tightly connected non-porous microvascular endothelial cells, a continuous basement membrane, and astrocyte foot processes around the basement membrane (Beggs et al., 2010). Cerebral microvascular endothelial cells are the anatomical basis of the blood–brain barrier, and the tight junction of the microvascular endothelium is the basis for maintaining the low permeability of the blood–brain barrier (Yeh et al., 2007). The presence of the blood–brain barrier prevents macromolecules from entering the brain tissue, and only small lipophilic molecules (<400 Da) can usually freely pass through the blood–brain barrier, which is important in maintaining the homeostasis of the brain environment (Persidsky et al., 2006). Studies have shown that cerebral ischemia–reperfusion can destroy the basement membrane of cerebral microvessels and make the tight connections between vascular endothelial cells disappear, thus damaging the blood–brain barrier. Increased permeability of the damaged blood–brain barrier leads to vasogenic brain edema and complicated cerebral hemorrhage, further aggravating brain injury (Yenari et al., 2006; Yeh et al., 2007). CGRP can reduce the overexpression of the aquaporin-4 (AQP-4) protein and its mRNA in the cerebral ischemic junction (Cai et al., 2010). At the same time, CGRP can also improve the ultrastructure of damaged microvascular endothelial cells and the damaged basement membrane, protecting the blood–brain barrier from destruction, thus reducing brain edema and protecting the damaged brain (Liu et al., 2011).

In conclusion, CGRP can protect the integrity of the blood–brain barrier and indirectly alleviate brain edema by reducing the overexpression of the AQP-4 protein and its mRNA.

### 5.3. Anti-inflammatory properties of CGRP after cerebral ischemia–reperfusion

**Figure 2:**
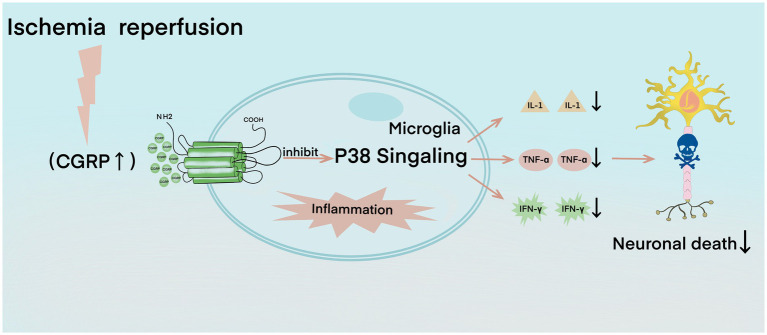
In the calcitonin gene-related peptide (CGRP)-mediated anti-inflammatory process, ischemia–reperfusion leads to the expression and activation of CGRP and then inhibits the p38 signaling pathway, resulting in the decline of interleukin-1, tumor necrosis factor-α, and interferon-γ, which further reduces neuronal death.

In the early stage of cerebral ischemia–reperfusion, small glial cells are activated, which induces the activation of the P38 signaling pathway that participates in the formation of free radicals and apoptosis and promotes the inflammatory response process (Piao et al., 2002). The P38 signaling pathway enhances the expression of pro-inflammatory factors and induces numerous inflammatory factors, such as tumor necrosis factor α (TNF-α), interleukin-1 (IL-1), and interferon-γ (IFN-γ), among which TNF-α, IL-1, and IFN-γ have harmful effects on ischemic brain tissue (Piao et al., 2003; Balaban et al., 2005). TNF-α recruits and activates leukocytes, resulting in increased leukocyte–endothelial cell adhesion, which then induces the expression of other cytokines, such as IL-1, IL-6, and IL-8, forming a positive feedback cycle and further aggravating brain injury (Fabry et al., 1992; Liu et al., 1994). CGRP can significantly reduce the expression level of phosphorylated P38, thus inhibiting the activation of the P38 signal pathway (Figure 2; Yang et al., 2016). CGRP is a key effector molecule of neurogenic inflammation. It reduces the activation of microglia, downregulates Th1 classical inflammation, and reduces the production of TNF-α, IL-2, and IFN-γ and the innate immune response of macrophages (Assas et al., 2014). CGRP also reduces the endothelial cell production of CCL2, which is also known as monocyte chemoattractant protein-1/MCP-1, and some other chemokines, such as CXCL1 and CXCL8 (Huang et al., 2011; Russell et al., 2014). CCL2 weakens the blood–brain barrier and attracts monocytes into brain tissue, whereas CXCL8 attracts neutrophils into the brain. Neutrophils destroy the endothelium during attachment, exacerbate reperfusion injury after ischemia, and increase the severity of local traumatic brain injury (Zarbock and Ley, 2008; Semple et al., 2010). CGRP has a protective effect on septic mice by restricting neutrophils and monocytes from blood vessels into the peritoneal cavity of mice (Gomes et al., 2005). CGRP also inhibits the chemotaxis of neutrophils in barrier tissues, such as the lungs and skin (Baral et al., 2018). Similarly, CGRP may protect the brain’s immunity by preventing leukocytes from crossing the blood–brain barrier (Semple et al., 2010). Additional supplementary explanations are provided by the CGRP gene knockout model; mice with a genetic lack of CGRP are characterized by increased inflammation, astrocyte activation, oxidative DNA damage, decreased expression of VEGF and insulin-like growth factor 1, and reduced new capillary compensation formation (Zhai et al., 2018). Similarly, in the animal model of multiple sclerosis, direct injection of CGRP into the cerebrospinal fluid seems to inactivate some microglia, leading to a reduction in neuroinflammation and disease severity (Sardi et al., 2014).

In addition, CGRP can significantly upregulate the expression of bFGF (Cai et al., 2010). Studies have shown that bFGF can reduce brain injury after global cerebral ischemia–reperfusion by downregulating the expression and activity of inflammatory factors TNF-α, IL-8, and IL-1 (Zhang et al., 2005). Furthermore, bFGF also downregulates the expression of vascular cell adhesion molecules stimulated by IL-1, resulting in a significant decrease in leukocyte recruitment and adhesion (Kikuchi, 2000; Tromp et al., 2000).

In conclusion, CGRP reduces the expression of inflammatory factors and chemokines and plays an anti-inflammatory role, thus protecting damaged brain tissues.

### 5.4. CGRP as a component of angiogenesis induction in ischemia–reperfusion injury

Angiogenesis is an important repair or protective mechanism in cerebral ischemic responses. Previous studies have found that hypoxia, ischemia, and a related increase in lactic acid and decrease in pH can induce the synthesis and release of CGRP (Wang et al., 1996; Wang and Fiscus, 1997). *In vivo*, CGRP increases angiogenesis during wound healing and tumor formation (Ohno et al., 2008; Toda et al., 2008a,b); these findings suggest that CGRP is involved in angiogenesis. Furthermore, the application of gepants, which is a CGRP receptor antagonist, continuously reduces the lateral circulation and the success rate of reperfusion, which increases the area of cerebral infarction and causes severe neurological dysfunction (Mulder et al., 2020). In most cases, CGRP binds to its type 1 receptor (i.e., CGRP R1) and initiates the cAMP–PKA pathway (Wimalawansa, 1996; Hutchinson et al., 2008). Previous studies have also suggested that CGRP may act as a local factor to stimulate the proliferation of endothelial cells and that the mechanism is related to the formation of cAMP (Haegerstrand et al., 1990). A study has further shown that the angiogenesis of CGRP is mediated by the CGRPR1–cAMP–AMP-activated protein kinase (AMPK)–eNOS signal cascade (Zheng et al., 2010). This study confirms that CGRP can activate AMPK *in vivo* and *in vitro*, whereas the pharmacological inhibition of CGRP and cAMP can weaken CGRP-activated AMPK *in vitro*. CGRP also induces the eNOS phosphorylation of HUVEC Ser1177 and Ser633 in a time-dependent manner, which can be blocked by the AMPK inhibitor compound C, which can also block the formation and migration of the HUVEC tube promoted by CGRP. AMPK promotes angiogenesis (Nagata et al., 2003), especially under hypoxic and/or ischemic stress (Shibata et al., 2004). In addition, AMPK indirectly promotes angiogenesis by stimulating endothelial progenitor cell (EPC) differentiation (Li et al., 2008).

Furthermore, CGRP-upregulated eNOS can further induce VEGF expression, which then promotes the survival and angiogenesis of vascular endothelial cells (Kang et al., 2013). Moreover, CGRP significantly increases the expression of VEGF receptors 1 (i.e., FLT) and 2 (i.e., KDR), whereas CGRP also enhances the expression of the CGRP1 receptor, thus inducing angiogenesis (Tuo et al., 2013). VEGF plays an important role in angiogenesis. VEGF can bind to VEGF receptors on the surface of vascular endothelial cells to activate various downstream signals, which induce the proliferation and differentiation of EPC and promote the proliferation, migration, and survival of endothelial cells (Li et al., 2017; Sun et al., 2020), thus stimulating angiogenesis and increasing the success rate of reperfusion. Notably, the effect of CGRP on VEGF may be time limited, as high levels of NO feedback reduce VEGF activity (Kimura and Esumi, 2003). This effect may also be protective because excessive VEGF signals can disrupt the blood–brain barrier (Dragoni and Turowski, 2018).

In addition, CGRP can significantly upregulate the expression of bFGF after ischemia–reperfusion injury (Cai et al., 2010). BFGF can promote angiogenesis and neurogenesis, thus improving the survival rate of neurons (Zhao et al., 2013). In fact, the application of bFGF increases the proliferation of neural progenitor cells after cerebral ischemia in adult rats (Türeyen et al., 2005). In adult SD rats, intravenous injection of bFGF can improve the score of neurological dysfunction and reduce the volume of cerebral infarction (Fisher et al., 1995; Li and Stephenson, 2002). The number of progenitor cells in the lateral ventricular subventricular zone of bFGF knockout mice has decreased by 50% (Zheng et al., 2004). Therefore, bFGF may play an important role in neurogenesis.

In conclusion, after ischemia–reperfusion injury, CGRP can increase the success rate of reperfusion by inducing neovascularization and accelerating the establishment of collateral circulation, thus promoting the survival and neurogenesis of neurons around cerebral infarctions and reducing the infarct area.

### 5.5. Antioxidant properties of CGRP within the brain

During cerebral ischemia–reperfusion, the restoration of blood flow supplements oxygen for tissues, and the increased production of oxygen free radicals further strengthens the oxidative stress of the brain. The clearance of ROS and production balance are disrupted, leading to signal transduction and cell damage induced by oxidative stress. Severe oxidative stress can lead to cell death through necrosis or apoptosis (Raedschelders et al., 2012; Karamyan, 2021). CGRP can protect endothelial cells from angiotensin II by reducing the production of oxidants and protecting antioxidant defense, thus maintaining the expression of eNOS (Smillie et al., 2014). The experiment has shown that CGRP activates the PI3K/Akt pathway and increases the expression of Nrf2 and HO-1, which decreases the levels of ROS and malondialdehyde, thus reducing apoptosis and oxidative stress (Liu et al., 2019). It can also prevent the injury and apoptosis of human umbilical vein endothelial cells induced by oxidized low-density lipoprotein (Luo et al., 2008).

These antioxidant processes may also have a protective effect on NO, increasing the utilization rate of brain-derived neurotrophic factors (BDNF). BDNF is the most abundant growth factor in the brain and plays an important role in antioxidant defense, protection of neurons from apoptosis and promotion of synaptic formation and plasticity, neurogenesis, and nerve repair (Marie et al., 2018). Notably, approximately 50% of BDNFs in brain tissues are produced in vascular endothelial cells, and the production of BDNF in endothelial cells is initiated by NO (Guo et al., 2008; Marie et al., 2018). CGRP can also increase the plasma level of BDNF and make trigeminal ganglion neurons secrete BDNF (Simonetti et al., 2008). Through these pathways, CGRP outside the blood–brain barrier can maintain and enhance the brain’s supply of BDNF and contribute to neuroprotection.

## 6. Current barriers

Although CGRP can play an important role in cerebral ischemia–reperfusion injury, the protective effect and prognosis of CGRP on ischemia–reperfusion injury still pose some challenges. First, current studies on the application of CGRP in ischemia–reperfusion injury are mainly focused on animal models, and whether it will produce the same effects in humans or be used in clinical adjuvant therapy remains to be further studied. At present, the model of transient middle cerebral artery occlusion (tMCAO) in rats is commonly used in cerebral ischemia–reperfusion model. The main methods are as follows: a thread of nylon monofilament line coated with poly-L-lysine was inserted along the internal carotid artery until resistance was sensed. After 2 h of cerebral ischemia, the nylon thread was removed and the wound was sutured. Subsequently, reperfusion was performed for 3 h. Following surgery, the rats were placed under an illuminating lamp to maintain the body temperature of the rats between 37 and 37.5°C (Yang et al., 2016; Du et al., 2018). The rats were treated with CGRP at the dose of 3 μg/kg (i.p.) at the beginning of reperfusion. Subsequently, rats were used for cerebral infarct volume determining, water content measurement, BBB permeability determining, neurobehavioral score evaluating and morris water maze test. The results showed that CGRP administration could significantly reduce infarct volume as well as postischemic increase of brain edema with a 2-h therapeutic window in the model of transient middle cerebral artery occlusion, so as to reduce the injury caused by cerebral ischemia–reperfusion (Liu et al., 2011). Second, whether adverse reactions or side effects of early intervention of exogenous CGRP in ischemic strokes exist have been scarcely reported and should be further evaluated. Third, whether the intervention of exogenous CGRP on ischemic stroke can maintain the long-term functional recovery of injured nerves remains to be further studied.

## 7. Conclusion and further perspectives

**Figure 3:**
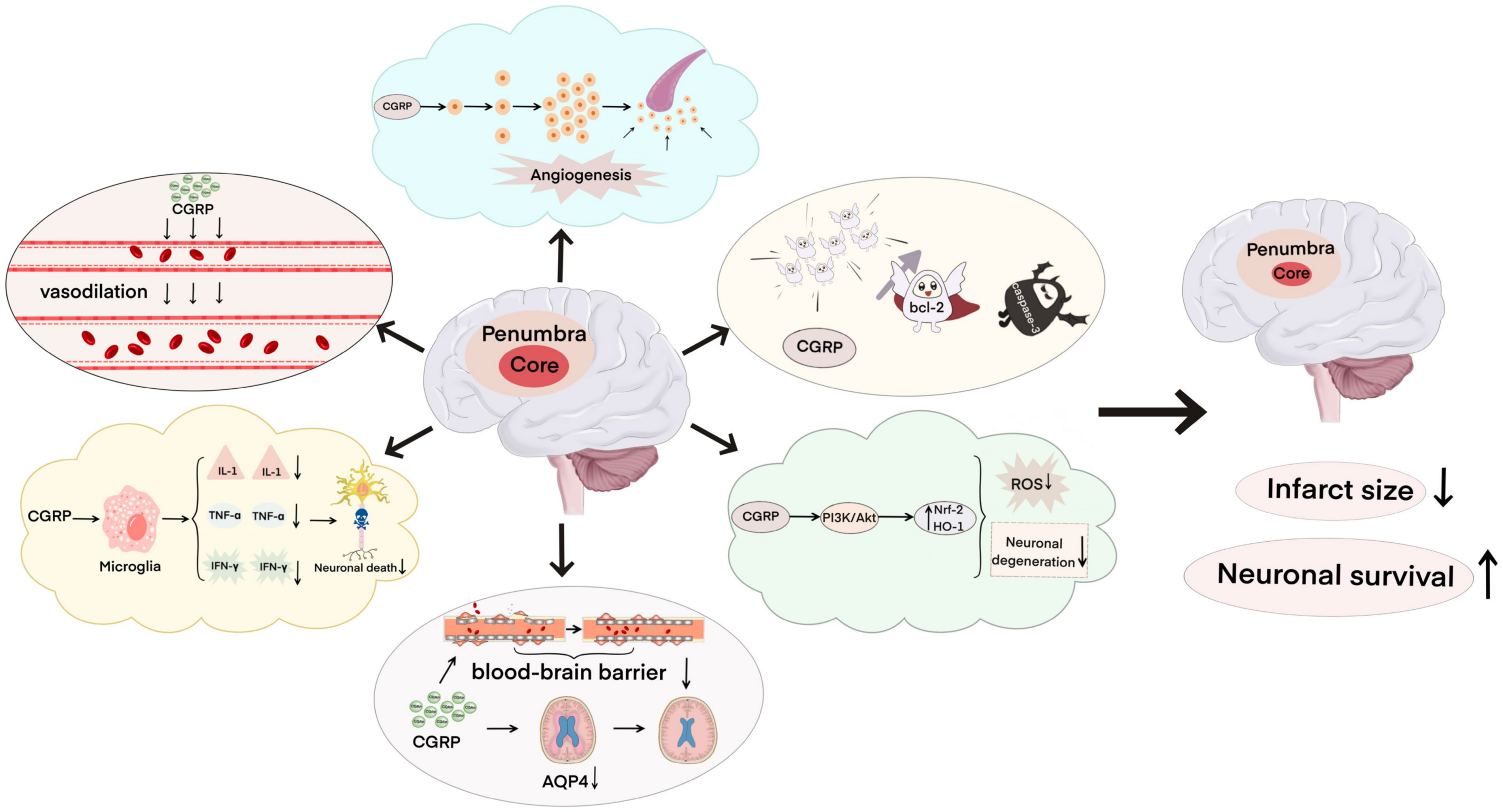
Neuroprotective effect of calcitonin gene-related peptide on cerebral ischemia–reperfusion injury, including dilating blood vessels, inhibiting the expression of inflammatory factors, relieving brain edema, resisting oxidative damage, inducing neovascularization, and inhibiting apoptosis, which reduces the infarct area and increases neuron survival.

CGRP and its signaling pathway-related proteins play a key role in cerebral ischemia–reperfusion injury. After cerebral ischemia–reperfusion, CGRP can bind to specific receptors to activate a series of signaling pathways, and CGRP can dilate blood vessels, inhibit the expression of inflammatory factors, reduce cerebral edema, fight oxidative injury, induce neovascularization, and inhibit apoptosis (Figure 3), thus reducing brain damage and repairing damaged nerve functions. It is a promising alternative drug for the intervention and treatment of ischemic strokes. I believe that in the near future, the development of molecular biology, pathology, and pharmacology can provide a great possibility for CGRP in preventing cerebral ischemia–reperfusion injury and a new direction for the treatment of ischemic stroke.

## Author contributions

JN and JX conceived and organized the writing of the manuscript. JX, ZW, and JB researched literature and wrote the manuscript. JX, ZW, and KC proofread the writing of the manuscript. All authors contributed to the article and approved the submitted version.

## Funding

This study was funded by the National Natural Science Foundation of China (Grant no. 82172531) and Natural Science Foundation of Fujian Province (Grant no. 2021J01709).

## Conflict of interest

The authors declare that the research was conducted in the absence of any commercial or financial relationships that could be construed as a potential conflict of interest.

## Publisher’s note

All claims expressed in this article are solely those of the authors and do not necessarily represent those of their affiliated organizations, or those of the publisher, the editors and the reviewers. Any product that may be evaluated in this article, or claim that may be made by its manufacturer, is not guaranteed or endorsed by the publisher.
